# Selenium nanoparticles for breast cancer: mechanisms, delivery strategies, and translational challenges

**DOI:** 10.3389/fonc.2026.1875872

**Published:** 2026-08-12

**Authors:** Huijia Ni, Xu Fu, Xiaoyu Chen, Lei Zhao

**Affiliations:** 1 The Second Clinical Medical College, Lanzhou University, Lanzhou, China; 2 Department of General Surgery, The Second Hospital of Lanzhou University, Lanzhou, China; 3 Emergency Center, The Second Hospital of Lanzhou University, Lanzhou, China; 4 Emergency Medicine Laboratory, The Second Hospital of Lanzhou University, Lanzhou, China; 5 Key Laboratory of Environmental Oncology of Gansu Province, The Second Hospital of Lanzhou University, Lanzhou, China

**Keywords:** breast cancer, nanomedicine, selenium nanoparticles, selenium-based agents, translational oncology

## Abstract

Breast cancer treatment remains limited by drug resistance, toxicity, and inadequate selectivity in aggressive subtypes, prompting continued interest in selenium-based agents whose anticancer activity depends strongly on chemical form, redox behavior, and metabolic fate. This review critically compares inorganic, organic, and nanoscale selenium, with particular emphasis on selenium nanoparticles (SeNPs) as engineerable platforms for breast cancer therapy. Current evidence indicates that selenium-based agents primarily exert antitumor effects through disruption of redox homeostasis, induction of apoptosis, and modulation of survival signaling pathways such as PI3K/AKT/mTOR, while additional effects on autophagy, metastasis, angiogenesis, and treatment resistance appear to be more context-dependent. Compared with conventional selenium compounds, SeNPs offer greater formulation flexibility through surface modification, cargo loading, and stimulus-responsive design, and have shown promising activity especially in triple-negative and HER2-positive breast cancer models. However, clinical translation is restricted by the lack of breast cancer-specific early-phase therapeutic studies and by persistent uncertainties regarding the therapeutic window, pharmacokinetics, long-term safety, manufacturing reproducibility, and patient selection. By integrating selenium speciation, SeNP preparation strategies, subtype-oriented applications, and translational barriers, this review highlights that future progress will depend on standardized formulations, rigorous *in vivo* validation, and biomarker-informed clinical development rather than unselected selenium use.

## 1 Introduction

Breast cancer is a heterogeneous disease, and treatment selection is guided by tumor stage, hormone receptor status, HER2 status, and molecular subtype. Although surgery, radiotherapy, chemotherapy, endocrine therapy, targeted therapy, and immunotherapy have improved clinical outcomes, treatment resistance, systemic toxicity, and limited durable therapeutic options remain important challenges, particularly in triple-negative breast cancer (TNBC) and treatment-resistant HER2-positive disease (1–7). In addition, inadequate tumor selectivity and intracellular drug delivery may restrict therapeutic efficacy while increasing off-target exposure (8, 9).

Selenium-based agents have attracted attention because their biological and anticancer effects depend strongly on chemical form, metabolic conversion, dose, and cellular redox context (10, 11). Inorganic selenium compounds such as sodium selenite can induce marked oxidative stress and tumor-cell death but are limited by a relatively narrow therapeutic window. Organic selenium compounds, including methylseleninic acid and selenomethionine, differ substantially in metabolic behavior and direct anticancer activity and should not be considered a pharmacologically uniform group (11–13). Selenium-based interventions should therefore be evaluated according to their specific chemical form rather than being discussed collectively as “selenium”.

Selenium nanoparticles (SeNPs) provide an additional level of complexity because their biological effects are influenced not only by the elemental selenium core but also by particle size, surface coating, charge, stabilizer, therapeutic cargo, and administration route (14–17). These formulation attributes enable SeNPs to function as engineerable platforms for drug loading, surface targeting, controlled release, and combination therapy. However, favorable preclinical activity does not necessarily indicate clinical readiness, because differences in formulation composition, manufacturing reproducibility, pharmacokinetics, biodistribution, long-term safety, and quality control remain insufficiently defined.

A recent review has summarized the general biological effects and therapeutic potential of selenium-containing compounds in breast cancer (18). The present review extends this literature by critically comparing sodium selenite, methylseleninic acid, selenomethionine, and SeNPs; evaluating SeNP synthesis, purification, physicochemical characterization, surface functionalization, and critical quality attributes; separating cell-based, animal, human observational, and clinical intervention evidence; and examining the principal barriers to breast cancer-specific clinical translation. Particular emphasis is placed on distinguishing nutritional selenium supplementation from pharmacological selenium compounds and therapeutic SeNP nanomedicines.

## 2 Review design and literature search

This review was conducted as a structured narrative review. PubMed/MEDLINE, Web of Science Core Collection, and Scopus were searched on 24 June 2026 for English-language publications formally published between 1 January 2005 and 30 April 2026. When electronic and issue dates differed, the earliest formal publication date was used. Search terms covered breast cancer, major selenium compounds, SeNP synthesis and characterization, drug delivery, toxicity, pharmacokinetics, biomarkers, and clinical translation. The complete database-specific search strategies are provided in **Supplementary Table 1**.

Eligible sources included *in vitro* and animal breast cancer studies, human observational and clinical studies, and publications addressing SeNP preparation, physicochemical properties, functionalization, delivery, safety, manufacturing, or translational development. Non-English articles, duplicate records, publications outside the predefined period, conference abstracts without sufficient data, editorials, corrections, retracted articles, and records with only incidental relevance were excluded. Studies involving other tumor types were considered only when they provided directly relevant formulation, pharmacokinetic, biodistribution, or safety information.

After database-specific and cross-database deduplication using PMID, DOI, and normalized titles, 1,932 unique records underwent title and abstract screening. Of these, 1,295 records were retained for further eligibility assessment, with full texts consulted as required. The final synthesis included 93 sources, comprising 91 peer-reviewed publications and two regulatory or technical documents. Cell-based, animal, human observational, and clinical evidence were evaluated separately. Because this was a structured narrative review, no formal risk-of-bias assessment, meta-analysis, or pooled effect estimation was performed.

## 3 Therapeutic limitations of current breast cancer treatment and the rationale for selenium nanoparticles

### 3.1 Limitations of current breast cancer therapies

Current breast cancer treatment includes surgery, radiotherapy, chemotherapy, endocrine therapy, targeted therapy, and, in selected settings, immunotherapy (5). Despite these advances, major limitations remain, including drug resistance, treatment-related toxicity, and insufficient durable options for aggressive subtypes such as triple-negative breast cancer (6, 7). In addition, suboptimal tumor selectivity and delivery efficiency may further restrict therapeutic benefit in some settings (8, 9).

### 3.2 Inorganic, organic, and nanoscale selenium: a comparative overview

Selenium-based agents comprise chemically and pharmacologically distinct categories whose biological effects depend on speciation, metabolic conversion, dose, and formulation characteristics (11). Inorganic selenium compounds, particularly sodium selenite, readily interact with intracellular thiol systems and can induce oxidative stress-mediated tumor-cell death. However, their nonspecific redox activity is associated with a relatively narrow and dose-dependent therapeutic window (12). Organic selenium compounds are also heterogeneous rather than uniformly low-toxicity agents. Methylseleninic acid (MSA) can generate pharmacologically active methylselenium species and has demonstrated apoptosis-promoting and chemosensitizing activity in preclinical breast cancer models, whereas selenomethionine (SeMet) is incorporated into proteins and functions mainly as a metabolically regulated selenium reservoir, with more limited direct antitumor activity (13, 19, 20).

SeNPs represent elemental selenium-based formulations whose biological behavior is determined not only by selenium itself but also by particle size, surface coating, charge, stabilizer, cargo, dose, and administration route (14–17, 21). Their principal distinction from soluble selenium compounds is therefore their engineering flexibility, including the capacity for stabilization, surface functionalization, and therapeutic cargo integration. However, SeNPs should not be regarded as uniformly more bioavailable or less toxic, because these properties remain formulation- and model-dependent. The pharmacological, safety, manufacturing, and translational differences among the major selenium-based agents are summarized in **Table 1**.

**Table 1:** Comparative pharmacological and translational characteristics of major selenium-based agents relevant to breast cancer.

Characteristics	Sodium selenite	Methylseleninic acid (MSA)	Selenomethionine (SeMet)	Selenium nanoparticles (SeNPs)
Chemical form	Inorganic selenium, Se(IV)	Organic methylselenium precursor	Organic selenoamino acid	Elemental selenium-based nanoscale formulation
Metabolic or intracellular processing	Reduced through thiol-dependent reactions to reactive selenium intermediates	Converted relatively directly to methylselenol-related metabolites	Incorporated nonspecifically into proteins and converted through additional metabolic steps	Cellular uptake and intracellular processing depend on particle size, coating, charge, stabilizer, and cargo
Principal anticancer activity	Redox disruption, oxidative stress, apoptosis, and context-dependent ferroptosis	Apoptosis induction, inhibition of survival signaling, and chemosensitization	Mainly serves as a selenium reservoir; comparatively limited direct cytotoxic activity	Selenium-related redox activity combined with targeting, cargo delivery, or stimulus-responsive release
Breast cancer evidence	Primarily cell-based studies, including MCF-7 and TNBC models; limited clinical safety data from mixed-cancer populations	Primarily preclinical breast cancer studies, including TNBC xenografts and chemotherapy-combination models	Limited breast cancer-specific therapeutic evidence; mainly nutritional and redox-related studies	Predominantly preclinical studies involving targeted, drug-loaded, and multimodal formulations
Typical administration or exposure context	Experimental oral or intravenous administration; *in vitro* exposure in mechanistic studies	Experimental administration in preclinical studies; no established breast cancer treatment route	Primarily oral nutritional supplementation	Formulation-dependent; intravenous administration is commonly evaluated in animal models, whereas many systems remain limited to *in vitro* testing
Tumor selectivity	No intrinsic tumor targeting	No intrinsic tumor targeting	No intrinsic tumor targeting	Targeting can be engineered through ligands, antibodies, peptides, aptamers, or coating materials
Bioavailability and exposure	Systemic exposure is dose- and route-dependent	Human pharmacokinetic data remain limited	Generally well absorbed orally and incorporated into body proteins	Strongly dependent on particle size, surface properties, colloidal stability, route of administration, and biological model
Major safety concerns	Narrow therapeutic window, nonspecific oxidative injury, and dose-dependent systemic toxicity	Dose- and exposure-dependent toxicity; limited long-term *in vivo* and clinical safety data	Generally tolerated at nutritional intake levels, but excessive or prolonged exposure may cause selenium toxicity	Potential aggregation, off-target accumulation, tissue retention, formulation-specific toxicity, and insufficient long-term safety data
Manufacturing and quality-control considerations	Conventional small-molecule preparation; chemical identity and dose can be defined reproducibly	Chemically defined compound, but stability and exposure conditions require control	Conventional pharmaceutical or nutritional manufacturing	Requires control of particle size, PDI, zeta potential, coating composition, selenium content, residual reagents, sterility, endotoxin, stability, and batch consistency
Current human evidence	Early-phase pharmacokinetic and safety studies mainly in mixed-cancer populations; no established breast cancer-specific efficacy	No established breast cancer-specific clinical efficacy	Human evidence mainly concerns supplementation and selenium status rather than anticancer treatment	No published breast cancer-specific therapeutic trial of a defined SeNP formulation
Principal translational limitation	Systemic toxicity and limited tumor selectivity	Limited *in vivo* validation and absence of breast cancer-specific clinical studies	Weak direct antitumor activity and lack of therapeutic evidence	Formulation heterogeneity, incomplete pharmacokinetic and safety characterization, manufacturing reproducibility, and lack of clinical validation
Representative references	(11, 12, 22–29)	(11, 12, 19, 30–32)	(11, 13, 19, 33–37)	(14, 15, 17, 20, 21, 38–45)

MSA, methylseleninic acid; PDI, polydispersity index; SeMet, selenomethionine; SeNPs, selenium nanoparticles; TNBC, triple-negative breast cancer.

The pharmacological and safety characteristics of SeNPs are formulation-, dose-, route-, and model-dependent. Findings obtained with one SeNP formulation should not be generalized to all SeNP-based systems.

### 3.3 Breast cancer-specific rationale for SeNP development

The relevance of SeNPs to breast cancer lies primarily in their potential to address delivery and selectivity limitations rather than to provide nonspecific selenium supplementation. Surface engineering and cargo integration allow selenium-related redox activity to be combined with chemotherapeutic agents, targeted drugs, or nucleic acid therapeutics in a single formulation (14–17, 21). Breast cancer-specific studies have further explored trastuzumab-, peptide-, aptamer-, and folate-functionalized SeNP systems for subtype-oriented delivery, particularly in HER2-positive and triple-negative breast cancer models (41–44). These strategies may be relevant to tumors characterized by drug resistance and inadequate intracellular delivery (6–9). Nevertheless, current evidence remains predominantly preclinical, and any proposed advantage should be demonstrated using appropriate comparisons with free drugs, unmodified SeNPs, and coating-, ligand-, or cargo-control formulations.

## 4 Preparation, formulation, and translational engineering of selenium nanoparticles

The biological activity and translational potential of selenium nanoparticles (SeNPs) are determined not only by the selenium core but also by the manufacturing process and the physicochemical properties of the final formulation. SeNP development therefore involves a sequential process comprising core synthesis, purification and stabilization, physicochemical characterization, surface functionalization or cargo integration, and manufacturing quality control (14–17, 21, 38–40, 45). This section evaluates these steps according to their effects on formulation reproducibility, biological performance, and translational feasibility.

### 4.1 Primary synthesis routes for SeNP production

SeNP cores are produced predominantly through chemical reduction or biological and green synthesis. These approaches differ in reaction controllability, particle-size distribution, residual impurities, batch reproducibility, purification requirements, and scale-up potential (14, 16, 17, 39, 40, 45–49). Regardless of the synthesis route, the resulting particles require subsequent purification, stabilization, and physicochemical characterization before biological evaluation or further functionalization.

#### 4.1.1 Chemical reduction methods

Chemical reduction is widely used because it is rapid, operationally simple, and adaptable to different stabilizers. Selenium precursors, most commonly sodium selenite, are reduced to elemental selenium using agents such as ascorbic acid, glutathione, or cysteine, while polymers, proteins, or polysaccharides are added to limit aggregation (14, 16, 21, 45, 50). Particle size and morphology depend on precursor-to-reductant ratio, pH, temperature, mixing conditions, reaction time, and stabilizer composition. Although this approach generally provides more controllable process parameters, residual precursors, reducing agents, and excess stabilizers must be adequately removed, and batch consistency must be demonstrated before scale-up (38–40, 45).

#### 4.1.2 Biological and green synthesis methods

Biological and green synthesis employs microorganisms, plant extracts, polysaccharides, or other naturally derived materials as reducing and stabilizing agents (15, 17, 46, 48, 51). These methods use relatively mild reaction conditions and may generate biomolecule-coated particle surfaces. However, differences in biological source, extract composition, precursor concentration, pH, temperature, and reaction time can produce substantial variation in particle size, morphology, and surface composition (15, 17, 46, 48). Biological residues, microbial contamination, endotoxin, and incompletely defined capping components may further complicate purification and quality control. Therefore, green synthesis should not be assumed to provide safer or more clinically suitable SeNPs without formulation-specific purity, reproducibility, stability, and safety assessment.

### 4.2 Purification, stabilization, and physicochemical characterization

Following synthesis, SeNP formulations should be purified to remove unreacted selenium precursors, reducing agents, excess stabilizers, and other soluble impurities. Common approaches include centrifugation with repeated washing, dialysis, and ultrafiltration, with the selected method depending on particle size, coating composition, and formulation stability (45, 52, 53). Polysaccharide-, protein-, or polymer-based coatings can reduce aggregation, although their stabilizing performance remains dependent on pH, ionic strength, temperature, and storage conditions (21, 52, 53).

Physicochemical characterization should distinguish core particle size and morphology measured by electron microscopy from hydrodynamic diameter and polydispersity index measured by dynamic light scattering. Zeta potential, selenium content, surface composition, colloidal stability, and selenium- or cargo-release behavior should also be reported when relevant (45, 52, 53). For biologically synthesized or injectable formulations, residual reagents, microbial burden, sterility, and endotoxin should be specifically evaluated (45, 54). These measurements are required because apparently similar SeNP formulations may exhibit different cellular uptake, biological activity, and toxicity when their physicochemical properties differ.

### 4.3 Surface functionalization and cargo integration

Bare SeNPs are prone to aggregation and generally lack intrinsic tumor specificity. Coating with polysaccharides, proteins, or polymers can improve colloidal stability and provide functional groups for further modification (21, 45, 52, 53). Targeting ligands and therapeutic cargos can then be incorporated to promote receptor-mediated uptake and controlled delivery (41–45). In breast cancer models, folate-, trastuzumab-, peptide-, and aptamer-functionalized SeNP systems have been explored for subtype-directed delivery (41–44). However, increasing structural complexity also increases the need to characterize ligand density, loading efficiency, release behavior, stability, purification, and batch reproducibility (38, 45, 54). Therefore, functionalized formulations should demonstrate a clear advantage over unmodified SeNPs using appropriate coating-, ligand-, and cargo-control groups.

### 4.4 Comparative manufacturing and translational suitability

Chemical reduction generally provides more controllable reaction parameters and greater potential for scale-up, but requires effective removal of unreacted selenium precursors, reducing agents, and excess stabilizers (14, 16, 39, 40, 45). Biological and green synthesis uses milder reaction conditions, but variability in biological sources and residual biomolecules may limit batch reproducibility and product standardization (15, 17, 46, 48). Surface functionalization can improve stability, targeting, and cargo delivery, but also increases formulation complexity and analytical requirements (21, 41, 42, 44, 45). No preparation method is universally superior; translational suitability depends on reproducible control of particle properties, purity, stability, sterility, batch consistency, and scalable manufacturing (38, 45, 54). The main manufacturing advantages and limitations of these preparation and engineering strategies are summarized in **Table 2**.

**Table 2:** Comparison of selenium nanoparticle preparation and engineering strategies relevant to translational development.

Preparation or engineering strategy	Particle-size control	Reproducibility	Scalability	Purification and safety challenges	Translational suitability
Chemical synthesis (14, 16, 39, 40)	Relatively controllable but sensitive to reaction conditions	Moderate to high after process optimization	Potentially scalable	Removal of unreacted precursors, reducing agents, and stabilizers is required	Suitable for defined manufacturing when process parameters are standardized
Biological or green synthesis (15, 17, 46, 48, 50, 51)	Often variable and source-dependent	Limited by biological-source and batch variability	Challenging	Biological impurities, endotoxin, and undefined metabolites may complicate purification	Requires extensive source, process, and quality standardization
Post-synthetic surface functionalization (21, 41, 45, 55)	Depends on the original SeNP core and modification process	May decrease with increasing formulation complexity	Formulation-dependent	Free ligands, crosslinkers, unloaded drugs, and coating instability require control	Improves targeting and cargo delivery but increases manufacturing and regulatory complexity

### 4.5 Critical quality attributes and translational manufacturing considerations

Translational development requires predefined critical quality attributes for the final SeNP product rather than characterization of particle formation alone. Core and hydrodynamic particle size, polydispersity index, zeta potential, morphology, selenium content, surface composition, and residual impurities should be included as basic quality attributes. Targeted or drug-loaded systems should additionally report coating identity, ligand density, drug-loading efficiency, free-drug content, release behavior, and stability in physiologically relevant media (38, 45, 54, 56, 57).

**Figure 1:**
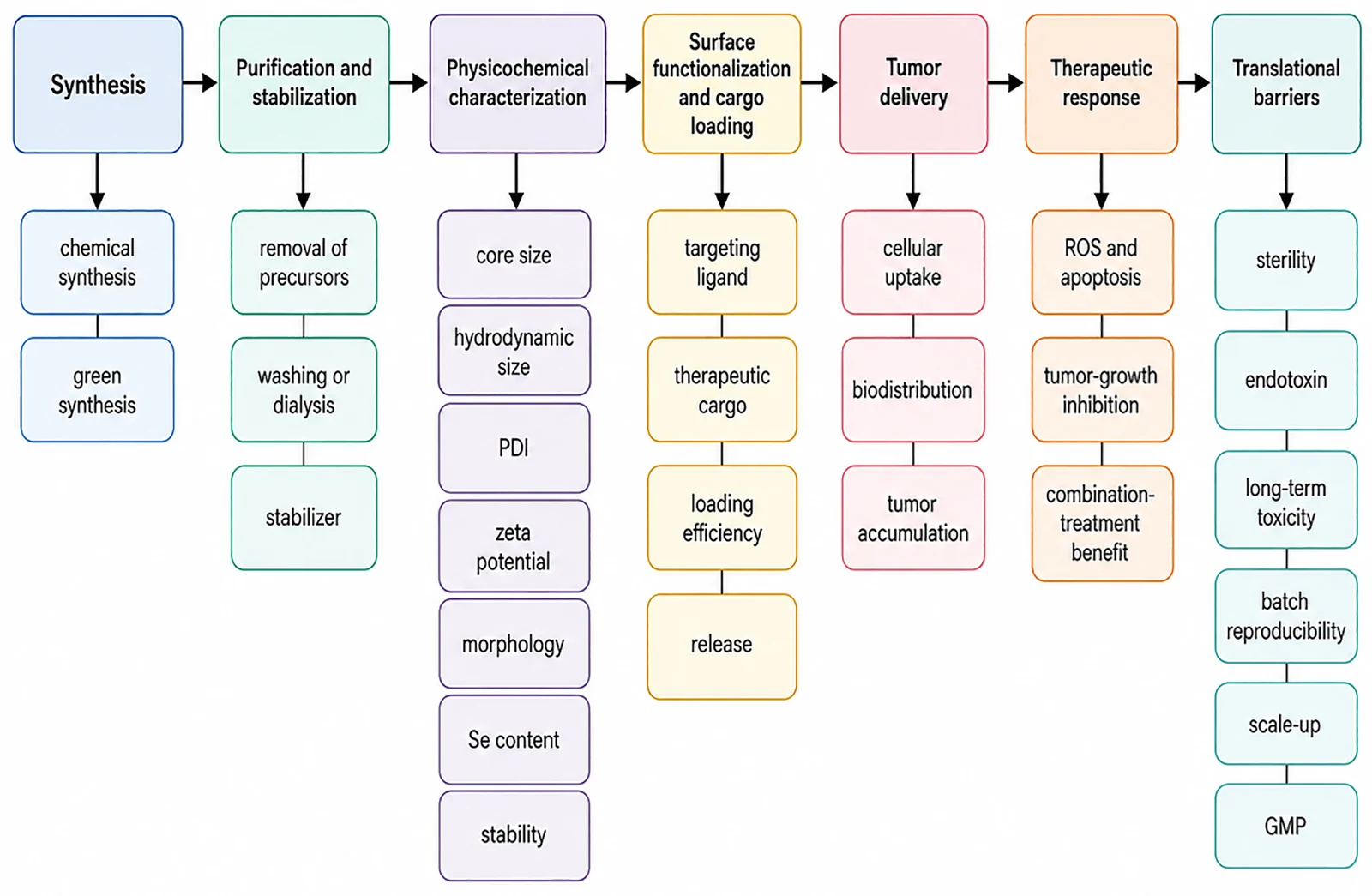
Integrated development pathway for selenium nanoparticle-based therapeutics from synthesis to clinical translation. Selenium nanoparticles (SeNPs) can be produced through chemical or green synthesis, followed by purification and physicochemical characterization, including assessment of particle size, polydispersity index (PDI), zeta potential, morphology, selenium content, and colloidal stability. Subsequent surface functionalization and cargo loading allow the incorporation of targeting ligands and therapeutic agents to support tumor delivery, biodistribution, and treatment response. Translational development requires concurrent evaluation of formulation stability, sterility, endotoxin contamination, long-term toxicity, batch-to-batch reproducibility, scalable manufacturing, and compliance with good manufacturing practice (GMP). The biological performance and translational suitability of each formulation remain dependent on particle composition, surface properties, dose, administration route, and experimental model.

Product stability should be evaluated by monitoring aggregation, particle size, PDI, zeta potential, selenium or cargo release, and biological activity during storage and after relevant processing steps such as lyophilization and reconstitution. Injectable products also require validated sterility and endotoxin control, while biologically synthesized formulations require additional control of source-related impurities (54, 56–59). Batch acceptance criteria and critical process parameters should be established before scale-up to ensure that changes in reaction volume, mixing, addition sequence, purification, or storage do not alter product performance. Because suitable specifications depend on formulation composition and administration route, results obtained from one SeNP preparation should not be generalized to all SeNP-based systems. The integrated development pathway of SeNP-based therapeutics, from core synthesis and formulation control to tumor delivery, therapeutic evaluation, and translational manufacturing, is summarized in **Figure 1**. The formulation characteristics and quality attributes reported for representative SeNP-based systems evaluated in breast cancer models are summarized in **Supplementary Table 2**.

## 5 Preclinical applications of selenium-based agents and SeNPs in breast cancer

Current evidence is derived mainly from breast cancer cell and animal models. Cell-based findings are interpreted as mechanistic evidence, whereas animal studies provide *in vivo* proof of concept rather than clinical evidence. Because selenium activity varies with chemical form, dose, metabolism, and formulation, findings for sodium selenite, methylseleninic acid (MSA), selenomethionine (SeMet), and selenium nanoparticles (SeNPs) should be interpreted separately (11, 19, 20, 60). Human evidence is discussed in Section 6.

### 5.1 Compound-specific pharmacology of selenium-based agents in breast cancer

Although redox disruption, apoptosis, and PI3K/AKT/mTOR modulation are frequently reported in studies of selenium-based agents, these effects do not represent an identical mechanism shared by all selenium compounds. Redox dysregulation is generally an upstream stress response, apoptosis is a downstream cell-death outcome, and PI3K/AKT/mTOR is a survival-signaling network that may be affected either directly or secondarily. The relative contribution of these processes depends on selenium speciation, metabolic conversion, dose, and formulation (11, 12, 19, 20).

#### 5.1.1 Sodium selenite

Sodium selenite is an inorganic Se(IV) compound with strong redox activity. Following cellular uptake, it reacts with intracellular thiol systems and is reduced to reactive selenium intermediates, thereby promoting reactive oxygen species generation and oxidative damage. In MCF-7 breast cancer cells, sodium selenite induced apoptosis through interconnected oxidative stress and endoplasmic reticulum stress, accompanied by p38 activation; antioxidant pretreatment attenuated these effects (12, 23). In triple-negative breast cancer cells, sodium selenite has also been reported to induce ATM-dependent ferroptosis, indicating that its redox activity may activate different cell-death programs according to cellular context (24). However, the same nonspecific redox reactivity that contributes to tumor-cell killing may also damage normal tissues, resulting in a relatively narrow and dose-dependent therapeutic window (11, 12).

#### 5.1.2 Methylseleninic acid

Methylseleninic acid (MSA) is an organoselenium compound that is readily reduced to methylselenol-related species without requiring the enzymatic cleavage needed by several selenoamino acids (31). This metabolic property allows MSA to exert relatively direct pharmacological effects on redox regulation and survival signaling. In breast cancer models, MSA has been reported to promote caspase-mediated apoptosis, reduce AKT-dependent survival signaling, and increase sensitivity to chemotherapeutic agents (30). In MCF-7 cells, MSA enhanced doxorubicin-induced apoptosis through suppression of phosphorylated AKT and modulation of downstream substrates (32). In MDA-MB-231 xenografts, MSA enhanced the antitumor activity of paclitaxel and increased apoptosis compared with either treatment alone (30). These findings support a chemosensitizing role for MSA, although its activity and toxicity remain dependent on dose, exposure duration, and tumor context.

#### 5.1.3 Selenomethionine

Selenomethionine (SeMet) is a selenium-containing analogue of methionine and differs metabolically from both sodium selenite and MSA. SeMet can be incorporated nonspecifically into general proteins in place of methionine and therefore functions partly as a stored selenium pool. Its conversion into more reactive selenium metabolites requires additional metabolic steps, which may limit its immediate pro-oxidant and cytotoxic activity (11, 61). In MCF-7 cells, SeMet modified antioxidant enzyme activity and cellular redox status, but comparatively high concentrations were required to produce marked oxidative or growth-inhibitory effects (37). SeMet should therefore not be assumed to have the same direct anticancer potency as sodium selenite or MSA. Its biological effects are more closely related to selenium availability, protein incorporation, exposure duration, and metabolic conversion than to rapid induction of oxidative cell death.

#### 5.1.4 Selenium nanoparticles

Selenium nanoparticles are predominantly composed of elemental selenium, but they should not be treated as a single pharmacologically uniform substance. Their cellular uptake, intracellular processing, redox behavior, and toxicity are strongly influenced by particle size, surface coating, charge, stabilizer, loaded cargo, and administered dose (14, 17, 40). In breast cancer models, SeNP formulations have been reported to induce oxidative stress, mitochondrial dysfunction, Bax/cytochrome c-related apoptosis, and changes in stress-associated signaling pathways (17, 62). One study showed greater SeNP-induced p38 activation and mitochondrial apoptosis in ERα-positive MCF-7 cells than in ERα-negative MDA-MB-231 cells, suggesting that cellular phenotype may influence SeNP sensitivity (62). Other functionalized SeNP systems have shown activity in drug-resistant breast cancer models or have combined selenium-related redox effects with drug delivery (63).

However, effects observed with coated or drug-loaded SeNPs cannot automatically be attributed to the elemental selenium core. The coating material, targeting ligand, or therapeutic cargo may independently contribute to reactive oxygen species generation, cellular uptake, or apoptosis. Appropriate controls for bare SeNPs, free coating materials, free drugs, and fully assembled formulations are therefore required before a selenium-specific mechanism can be assigned.

#### 5.1.5 Integrated redox, mitochondrial, apoptotic, and ferroptotic signaling

Selenium-based agents initiate different metabolic events but may converge on redox-sensitive cell-death pathways. Sodium selenite undergoes thiol-dependent reduction, MSA generates methylselenol-related metabolites, and SeNP activity depends on cellular uptake and formulation properties. These processes may increase reactive oxygen species (ROS), activate stress signaling, and suppress survival pathways such as AKT, NF-κB, and JAK2/STAT3 (11, 12, 19, 20, 23, 32, 64, 65).

Excessive ROS can promote mitochondrial membrane depolarization, Bax/cytochrome c signaling, and caspase-dependent apoptosis. Sodium selenite induced oxidative and endoplasmic reticulum stress in MCF-7 cells and activated the Bax–caspase axis in 4T1 cells, whereas MSA enhanced apoptosis through inhibition of AKT- and JAK2/STAT3-related signaling (23, 32, 64, 65). Selected SeNP formulations have also induced mitochondrial apoptosis, although effects from the selenium core, coating, and cargo should be distinguished (17, 62).

**Figure 2:**
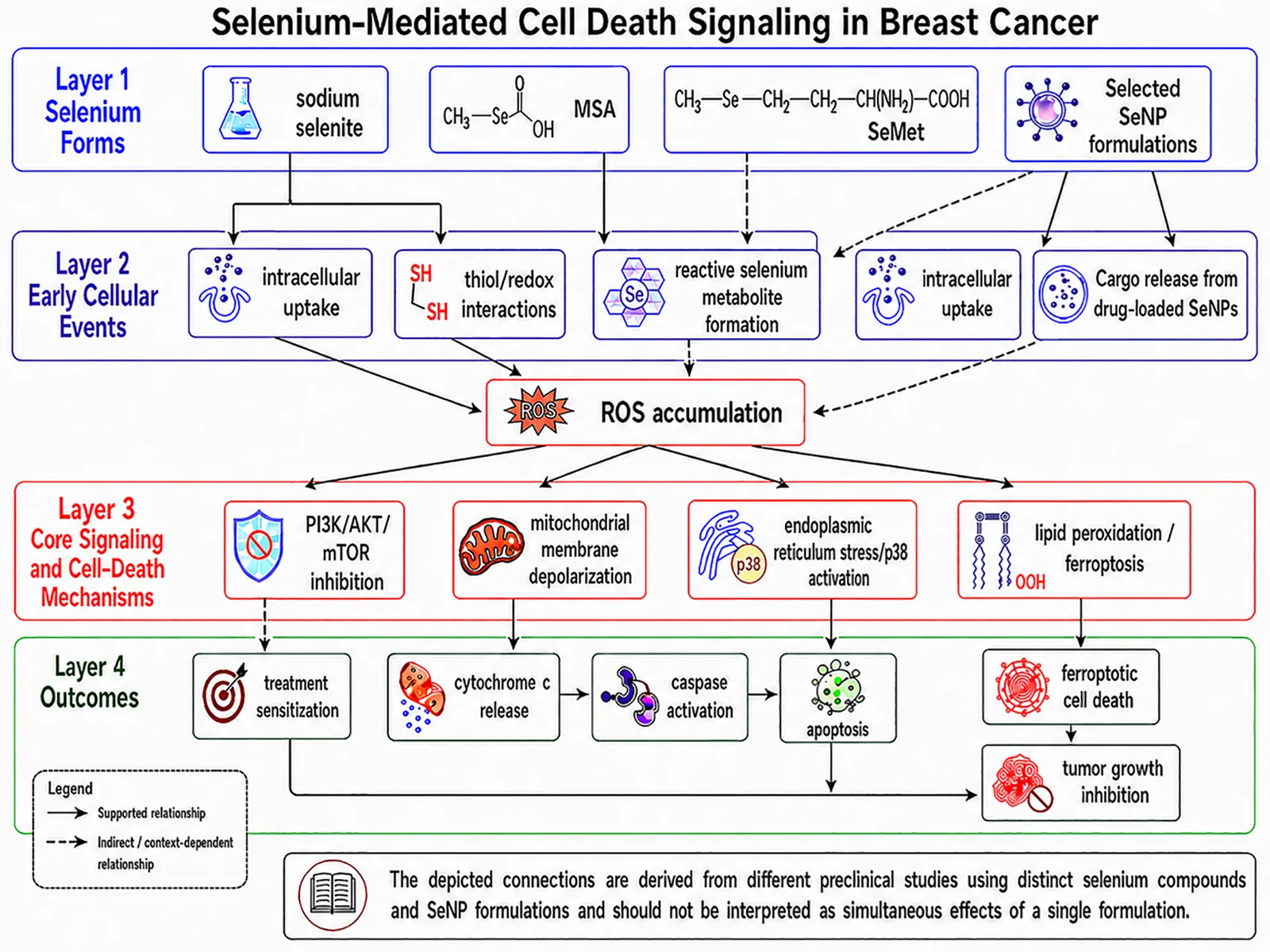
Integrated selenium-mediated cell death signaling in breast cancer. Distinct selenium forms and selected SeNP formulations may converge on ROS accumulation, PI3K/AKT/mTOR inhibition, mitochondrial dysfunction, endoplasmic reticulum stress, apoptosis, and ferroptosis, ultimately contributing to treatment sensitization and tumor growth inhibition. Solid arrows indicate supported relationships, whereas dashed arrows indicate indirect or context-dependent relationships. The depicted relationships were derived from different preclinical studies using distinct selenium compounds and SeNP formulations and should not be interpreted as simultaneous effects of a single formulation.

Sodium selenite additionally induced ATM-dependent ferroptosis in MDA-MB-231 cells, characterized by lipid peroxidation, glutathione depletion, iron accumulation, and altered GPX4 and FTH1 expression (24). This mechanism should not be generalized to all selenium compounds or SeNP formulations. The final response depends on selenium form, dose, formulation, tumor subtype, and cellular antioxidant capacity. These compound- and context-dependent mechanisms are summarized in **Figure 2**.

### 5.2 Selenium nanoparticles as formulation-dependent drug delivery platforms

The principal advantage of selenium nanoparticles (SeNPs) in breast cancer is their capacity to integrate selenium bioactivity with drug loading, surface targeting, and controlled release. However, the biological performance of each system depends on its particle composition, coating, cargo, and targeting ligand; therefore, different SeNP formulations should not be regarded as pharmacologically equivalent (38–40).

#### 5.2.1 Delivery of chemotherapeutic agents

SeNP-containing nanocarriers have been developed to improve the intracellular delivery and antitumor activity of chemotherapeutic agents. Hyaluronic acid-functionalized selenopolymeric nanocarriers enhanced doxorubicin-induced apoptosis in MCF-7 cells and inhibited three-dimensional tumor-sphere growth (66). A Se@Au@mSiO_2_ platform further combined doxorubicin delivery with photothermal therapy, resulting in increased oxidative stress and cytotoxicity in breast cancer cells (67, 68). In addition, selenium-containing prodrug nanoparticles have enabled the codelivery of bortezomib and selenium and suppressed tumor growth in a 4T1 mouse model (69). Nevertheless, these formulations differ substantially in structure and release behavior, limiting direct comparison between studies.

#### 5.2.2 Delivery of targeted agents and nucleic acids

In HER2-positive breast cancer, trastuzumab-functionalized albumin-coated hollow mesoporous SeNPs have been used to deliver pyrotinib and improve HER2-directed treatment (41). In triple-negative breast cancer, peptide-modified SeNPs protected Src-targeting small interfering RNA, enhanced its cellular uptake, and reduced Src expression in MDA-MB-231 cells (42). These studies support the adaptability of SeNPs for subtype-specific delivery, although the siRNA evidence remains primarily cell-based.

#### 5.2.3 Stimuli-responsive and multimodal systems

Stimuli-responsive SeNP systems have been designed to release their therapeutic components under tumor-associated conditions. An aptamer-modified, glutathione-responsive SeNP platform enhanced uptake and cytotoxicity in MDA-MB-231 cells (44). Other selenium-containing systems have combined chemotherapy with photothermal therapy, imaging, radiotherapy, or immunotherapy (70). However, elemental SeNPs, SeO_2_ nanoparticles, and diselenide-containing polymeric carriers are chemically distinct and should be clearly differentiated. Their efficacy should also be evaluated using controls for the free drug, unloaded carrier, bare SeNPs, and complete formulation.

### 5.3 Subtype-specific applications in breast cancer

Breast cancer subtypes, including TNBC, HER2-positive, and ER+/HER2-negative disease, may respond differently to selenium-based agents because of their distinct molecular features (7). Among these subtypes, current preclinical evidence is relatively stronger for TNBC and HER2-positive breast cancer, whereas evidence in ER+/HER2-negative disease remains limited (7, 24, 41, 42, 44, 71).

#### 5.3.1 Triple-negative breast cancer

In TNBC models, sodium selenite induced ATM-dependent ferroptosis through redox disruption and changes in ferroptosis-related proteins (24). Methylseleninic acid also enhanced paclitaxel activity by promoting apoptosis and cell-cycle arrest (30). In addition, targeted SeNP systems have been explored for siRNA delivery, redox-responsive treatment, and multimodal therapy (42, 44, 67). SELENOS and VCP/p97 have been proposed as potential prognostic-associated markers in TNBC, but their predictive value for selenium treatment has not been established (71).

#### 5.3.2 HER2-positive breast cancer

In HER2-positive models, selenium-conjugated trastuzumab showed cytotoxic activity in trastuzumab-sensitive and trastuzumab-resistant breast cancer cells (72). Trastuzumab-functionalized hollow mesoporous SeNPs have also been developed to improve pyrotinib delivery (41). However, these findings remain preclinical, and no therapeutic SeNP trial has established efficacy in patients with HER2-positive breast cancer.

#### 5.3.3 ER+/HER2-negative breast cancer

Evidence in ER-positive/HER2-negative breast cancer remains limited. A dihydroselenoquinazoline inhibited S6 ribosomal protein signaling, induced apoptosis, and suppressed autophagy in MCF-7 cells (73). Methylselenocysteine combined with tamoxifen also inhibited MCF-7 xenograft growth through increased apoptosis and reduced angiogenesis (74). Nevertheless, these findings are based on a small number of preclinical studies and require validation in more representative endocrine-resistant models.

### 5.4 Selenium-based strategies in combination therapy

Combination therapy is one of the most clinically relevant directions for selenium-based strategies in breast cancer. Current preclinical evidence suggests that selenium compounds and SeNP-based systems may enhance the effects of chemotherapy, radiotherapy, and targeted therapy through chemosensitization, redox modulation, improved drug delivery, or reversal of treatment resistance (30, 63, 72, 75–77). However, the available evidence ranges from cell-based studies to animal models, and the clinical value of these combinations remains unestablished.

#### 5.4.1 Combination with chemotherapy

Methylseleninic acid (MSA) enhanced paclitaxel-induced apoptosis and tumor inhibition in triple-negative breast cancer models (30) and sensitized MCF-7 cells to doxorubicin through suppression of AKT-related survival signaling (32). Nano-formulated selenium has also been evaluated with doxorubicin in resistant breast cancer cells (63). In addition, fish oil and selenium supplementation enhanced the antitumor activity of doxorubicin in a TNBC mouse model (76). Selenium combined with docetaxel produced synergistic growth inhibition in MDA-MB-231 cells, but not in MCF-7 cells, indicating a cell-dependent response (78).

#### 5.4.2 Combination with radiotherapy

SeNPs have been reported to enhance radiation-induced breast cancer cell death through oxidative stress, G2/M arrest, and autophagy (77). By contrast, clinical studies of conventional selenium supplementation during radiotherapy have mainly focused on reducing treatment-related toxicity rather than increasing tumor radiosensitivity (79–81). These two applications should therefore be clearly distinguished.

#### 5.4.3 Combination with targeted therapy

In HER2-positive breast cancer cells, selenium-conjugated trastuzumab showed activity in both trastuzumab-sensitive and resistant models (72). Trastuzumab-functionalized SeNPs have also been developed to deliver pyrotinib (41). In TNBC-bearing mice, fish oil and selenium enhanced the antitumor effect of bevacizumab (75), whereas methylselenocysteine combined with tamoxifen inhibited MCF-7 xenograft growth through increased apoptosis and reduced angiogenesis (74). Further studies are required to determine the contribution of selenium relative to the combined drug or delivery system. Representative preclinical studies are summarized in **Table 3** to facilitate comparison of formulation characteristics, experimental models, therapeutic outcomes, and reported toxicity.

**Table 3:** Representative preclinical studies of selenium-based nanoplatforms in breast cancer.

Formulation	Experimental model	Size (nm)	Cargo/targeting	Main outcome	Toxicity finding	Ref.
Biogenic SeNPs	4T1, MCF-7; no animal model	78–79	None	Selective cytotoxicity	Normal-cell toxicity detected	(17)
HBHSEP	SK-BR-3, MCF-7; tumor-bearing mice	172 ± 3.4	Pyrotinib; trastuzumab	HER2 targeting; apoptosis; tumor inhibition	NR	(41)
LP5-SeNP/siRNA	MDA-MB-231; no animal model	67*	Src siRNA; peptide	Src silencing; enhanced uptake	No cytotoxicity at tested dose	(42)
FA@SeNPs	4T1; 4T1 tumor-bearing mice	~60	Folic acid	Higher cytotoxicity; reduced tumor growth	NR	(43)
Se@MONs@LXL-1	MDA-MB-231, MCF-7, MCF-10A; no animal model	80 ± 15†	LXL-1 aptamer; GSH response	Enhanced uptake; cytotoxicity	NR	(44)
Alginate-SeNPs	MCF-7, MDA-MB-231; DMBA-treated rats	40–90	None	ERα-linked apoptosis; tumor inhibition	NR	(62)
Se@CMHA-DOX	MCF-7; 3D spheroids	NR	DOX; HA	TrxR inhibition; apoptosis; spheroid inhibition	NR	(66)
Se@Au@mSiO_2_/DOX	MCF-7, MDA-MB-231; MDA-MB-231 xenografts	~100	DOX; NIR	MDR reversal; tumor regression	No major organ toxicity	(67)
Se@β-CD-FA@PTX	MCF-7, MCF-10A; MCF-7 xenografts	70	PTX; folate	ROS-mediated apoptosis; tumor inhibition	Stable body weight	(68)
Se_BSAL-HP NPs	4T1; 4T1 tumor-bearing mice	204.13 ± 0.02	Bortezomib; sialic acid	Marked tumor inhibition	NR	(69)
Nano-Se plus irradiation	MCF-7; no animal model	NR	Radiotherapy	ROS; G2/M arrest; autophagy	NR	(77)

β-CD, beta-cyclodextrin; DMBA, 7,12-dimethylbenz[a]anthracene; DOX, doxorubicin; GSH, glutathione; HA, hyaluronic acid; HER2, human epidermal growth factor receptor 2; MDR, multidrug resistance; MON, mesoporous organosilica nanoparticle; NIR, near-infrared; NR, not reported or not clearly specified; PTX, paclitaxel; SeNPs, selenium nanoparticles; siRNA, small interfering RNA,CMHA, cetyl-modified hyaluronic acid; FA, folic acid; HBHSEP, trastuzumab-functionalized albumin-coated hollow mesoporous selenium nanoparticles loaded with pyrotinib; NPs, nanoparticles; ERα, estrogen receptor alpha; TrxR, thioredoxin reductase.

Particle size represents the final formulation or main particle population reported in each study. *Size before siRNA complexation. †Dry particle size. Numerical PDI values were not clearly reported in most studies and were therefore omitted from the table.

The available studies differ substantially in formulation design, experimental models, and outcome assessment. Limited reporting of particle dispersity and toxicity restricts direct comparison and assessment of translational readiness.

### 5.5 Influence of the breast tumor microenvironment on SeNP performance

The breast tumor microenvironment may strongly influence the delivery and activity of selenium nanoparticles (SeNPs). Hypoxia, extracellular acidity, altered redox balance, and high intracellular glutathione can affect particle stability, selenium processing, cargo release, and ROS-dependent cytotoxicity. Glutathione-responsive SeNP systems may exploit the reducing intracellular environment for triggered release; however, abundant glutathione may also buffer oxidative stress and reduce selenium-induced cell death. These effects are likely to depend on coating composition, particle size, dose, and tumor subtype (38, 39, 41, 44, 52, 53).

Abnormal tumor vessels, heterogeneous perfusion, elevated interstitial pressure, and a dense extracellular matrix can restrict SeNP extravasation and penetration into poorly vascularized regions. In breast tumor spheroid models, increasing fibroblast and collagen content reduced nanoparticle penetration, indicating that results obtained in monolayer cultures may overestimate delivery efficiency (82, 83). After systemic administration, adsorption of plasma proteins can generate a protein corona that changes surface identity, masks targeting ligands, and alters cellular uptake (84). Systemically administered nanoparticles may be sequestered by monocytes and tumor-associated macrophages, although this effect has not been adequately validated for SeNPs (85).

Target expression is another source of variability. HER2-, folate receptor-, peptide-, aptamer-, and CD44-directed SeNP systems have shown enhanced uptake in selected breast cancer models (41–44, 66), but receptor expression can vary between patients, tumor regions, and treatment stages. Consequently, efficacy observed in receptor-high cell lines may not be reproduced in heterogeneous tumors. Future studies should therefore assess target density, protein-corona formation, stromal penetration, immune-cell uptake, and spatial biodistribution using three-dimensional cultures, orthotopic or immunocompetent models, and patient-derived systems. Appropriate comparison with bare SeNPs, non-targeted particles, and free cargo is also required to determine whether the microenvironment-responsive design provides a genuine therapeutic advantage.

Much of the current understanding is extrapolated from general nanomedicine or non-breast tumor models, and breast cancer-specific validation remains limited.

## 6 Human evidence, translational barriers, and future directions

Human evidence concerning selenium should be interpreted according to its chemical form, dose, administration route, and clinical purpose. Nutritional supplementation, supportive care, pharmacological selenium compounds, and SeNP-based nanomedicines are distinct interventions and should not be considered interchangeable.

### 6.1 Current status of human evidence

Epidemiological studies have reported inconsistent associations between selenium status and breast cancer risk, and current evidence does not support routine preventive supplementation in unselected populations (33, 34). The SELECT trial also failed to demonstrate a general cancer-preventive benefit of selenomethionine, although its findings cannot be directly extrapolated to therapeutic selenium compounds or SeNPs (35, 36).

In breast cancer, most clinical evidence concerns supportive care rather than direct antitumor treatment. Selenium status has been evaluated during radiotherapy, and sodium selenite has shown possible benefit in breast cancer-related lymphedema (86, 87). However, these studies do not establish improved tumor control, and unnecessary supplementation may increase the risk of excessive selenium exposure (88).

Pharmacological selenium compounds have been evaluated mainly in mixed cancer populations. Studies of sodium selenite and other selenium compounds provide preliminary pharmacokinetic, pharmacodynamic, and safety information (25–29), but do not establish breast cancer-specific efficacy. More importantly, no published breast cancer-specific early-phase therapeutic trial of a defined SeNP formulation was identified. Therefore, evidence from supplementation or soluble selenium compounds should not be used as direct evidence for SeNP efficacy or safety. The distinct categories of human evidence and their appropriate interpretation are summarized in **Table 4**.

**Table 4:** Distinct categories of human evidence for selenium-related interventions in breast cancer.

Evidence category	Selenium exposure or intervention	Current evidence	Appropriate interpretation
Endogenous selenium status	Circulating or tissue selenium	Associations with breast cancer risk and prognosis have been reported, but results remain heterogeneous (33, 34, 89)	Observational and hypothesis-generating; does not establish causality
Preventive supplementation	L-selenomethionine in SELECT	No cancer-preventive benefit in the tested population (35, 36)	Does not support generalized supplementation; not direct evidence for SeNP therapy
Supportive care	Selenium status or sodium selenite during breast cancer treatment	Limited evidence concerning radiotherapy toxicity and lymphedema-related outcomes (79–81, 86–88)	Possible supportive role; not evidence of direct antitumor efficacy
Pharmacological selenium compounds	Oral or intravenous sodium selenite and other defined selenium compounds	Early-phase pharmacokinetic, pharmacodynamic, and safety data are available mainly from mixed cancer populations (25–29)	Provides dose and safety context but does not establish breast cancer-specific efficacy
Biomarker-associated evidence	Blood selenium, SELENOP, GPX3	Associations with pCR, recurrence, mortality, or prognosis have been reported (89, 90)	Supports prospective biomarker research; not validated for treatment selection
SeNP-based nanomedicines	Defined therapeutic SeNP formulations	Breast cancer evidence remains predominantly preclinical	Requires formulation-specific manufacturing, pharmacology, safety, and early-phase clinical evaluation

GPX3, glutathione peroxidase 3; pCR, pathological complete response; SeNPs, selenium nanoparticles; SELENOP, selenoprotein P.

Observational associations with selenium status or selenoproteins do not establish causality or demonstrate that selenium supplementation or SeNP treatment improves clinical outcomes.

### 6.2 Major translational barriers to clinical application

Clinical translation of SeNPs remains limited by formulation-dependent pharmacokinetics, biodistribution, degradation, tissue retention, off-target exposure, and toxicity. Particle size, polydispersity, surface charge, coating composition, therapeutic cargo, dose, and administration route may substantially alter biological performance. Therefore, safety or efficacy findings obtained with one SeNP formulation should not be generalized to other systems (38–40, 45, 52, 53, 59, 91, 92).

Reproducible production is another major challenge. Variations in precursor concentration, reduction conditions, purification, stabilizer composition, ligand density, and cargo loading may affect particle characteristics, release behavior, and biological activity. Independent batches should therefore demonstrate consistent particle size, selenium content, surface properties, loading efficiency, release profile, stability, and therapeutic potency (38, 45, 52, 53, 57, 58). Increasing formulation complexity may also raise the costs of raw materials, purification, characterization, storage, and quality control, while the cost-effectiveness of SeNP-based breast cancer therapy remains unknown (91).

Preclinical studies should include appropriate comparisons with free drug, bare SeNPs, unloaded carriers, coating materials, and complete formulations. Subtype-relevant animal models should be used to evaluate dose–response relationships, pharmacokinetics, tumor accumulation, organ distribution, degradation, and long-term toxicity. Current breast cancer evidence remains predominantly preclinical, and the safety or therapeutic effects of soluble selenium compounds and nutritional supplementation should not be extrapolated directly to defined SeNP formulations.

### 6.3 Biomarker-guided development of selenium-based strategies

Biomarker-guided development may help address variability in selenium response. Pretreatment whole-blood selenium levels have been associated with pathological complete response after neoadjuvant chemotherapy, particularly in patients with HER2-positive or triple-negative breast cancer (90). However, this observational association does not demonstrate that selenium supplementation improves treatment response.

Serum selenium, selenoprotein P, and glutathione peroxidase 3 have also been associated with recurrence and mortality after breast cancer diagnosis (89). In addition, matched analysis of circulating selenium and the breast cancer selenotranscriptome suggests that systemic selenium status may modify the prognostic associations of specific tumor selenoproteins (93). Preclinical candidates such as SELENOS and VCP/p97 may provide further subtype-relevant signals in triple-negative breast cancer (71).

Nevertheless, none of these markers has been prospectively validated for selecting patients for selenium treatment. Terms such as “predictive biomarker” should therefore be avoided at this stage. Future trials should prospectively assess baseline selenium status, selenium speciation, tumor subtype, pharmacodynamic responses, and formulation-specific systemic exposure.

### 6.4 Future research priorities

Future research should prioritize formulation-specific evaluation rather than treating SeNPs as a uniform therapeutic class. Preclinical studies should include bare SeNPs, free drugs, unloaded carriers, coating materials, targeting ligands, and complete formulations to distinguish selenium-related effects from those of the cargo or surface modification. Candidate formulations should also demonstrate reproducible particle properties, physiological stability, and a favorable efficacy–toxicity balance before further development (38, 45, 52–54, 57–59).

Validation in clinically relevant breast cancer models is equally important. Three-dimensional cultures, orthotopic tumors, immunocompetent models, and patient-derived systems should be used to assess tumor penetration, biodistribution, treatment response, and long-term toxicity. Triple-negative and HER2-positive breast cancers remain rational initial settings because current preclinical evidence is relatively stronger in these subtypes (7, 41, 42, 44, 71).

Finally, biomarker development should move from retrospective association to prospective validation. Future studies should evaluate baseline selenium status, selenium speciation, tumor subtype, target expression, and pharmacodynamic responses. Biomarker-guided patient selection should be considered only after these factors have been shown to predict the response to a defined selenium compound or SeNP formulation (89, 90).

## 7 Conclusions

Selenium-based agents constitute a chemically and pharmacologically heterogeneous group, and sodium selenite, methylseleninic acid, selenomethionine, and SeNPs should not be regarded as interchangeable interventions. Current evidence for their antitumor activity in breast cancer remains predominantly derived from cell-based and animal studies, while human evidence mainly concerns selenium status, supplementation, supportive care, or soluble selenium compounds rather than therapeutic SeNP nanomedicines. SeNPs offer valuable engineering flexibility for targeting, cargo delivery, and combination treatment, but their efficacy, toxicity, and biodistribution remain dependent on formulation composition, dose, administration route, and experimental model. Clinical translation will require standardized and reproducible formulations, rigorous pharmacokinetic and long-term safety evaluation, appropriate formulation controls, and breast cancer-specific early-phase trials incorporating prospective biomarker assessment.
